# Draft Genome Sequence of *Salmonella enterica* subsp. *enterica* Serovar Putten Strain CRJJGF_00159 (Phylum *Gammaproteobacteria*)

**DOI:** 10.1128/genomeA.00895-16

**Published:** 2016-09-01

**Authors:** Sushim K. Gupta, Elizabeth A. McMillan, Charlene R. Jackson, Prerak T. Desai, Steffen Porwollik, Michael McCleland, Lari M. Hiott, Shaheen B. Humayoun, Jonathan G. Frye

**Affiliations:** a Bacterial Epidemiology and Antimicrobial Resistance Research Unit, USDA-ARS, Athens, Georgia, USA; b Department of Microbiology, University of Georgia, Athens, Georgia, USA; c Department of Microbiology and Molecular Genetics, University of California Irvine, Irvine, California, USA

## Abstract

Here, we report a 4.90 Mbp draft genome sequence of *Salmonella enterica* subsp. *enterica* serovar Putten strain CRJJGF_00159 isolated from food animal in 2004.

## GENOME ANNOUNCEMENT

*Salmonella* is one of the major zoonotic food-borne pathogens representing an important public health concern worldwide. This microbe causes several clinical manifestations and is responsible for many outbreaks of human salmonellosis (1, 2). About, 14.4% of 12,350 reported human infections from 1999 to 2003 were caused by *Salmonella* serotypes that also occurred in feed in Denmark (3). A high prevalence of *S.* Putten have been recorded from swine farms in Canada (4) and, animal feed material in Sweden (5).

Standard microbiology techniques were applied to isolate *S.* Putten strain CRJJGF_00159 from a food animal. The isolate was serotyped using SMART typing (6), and sequencing reads were used to determine antigenic formula to predict the Serotype using SeqSero (7), which predicted the antigenic formula of 13:d:1,w, designated Putten. Susceptibility testing for the strain was performed using broth microdilution plates for the Sensititre semi-automated antimicrobial susceptibility system (TREK Diagnostic Systems, Inc., Westlake, OH). Results were interpreted according to Clinical and Laboratory Standards Institute (CLSI) guidelines (8).

The genomic DNA was isolated from the overnight culture using the GenElute bacterial genomic DNA kit (Sigma-Aldrich, St. Louis, MO) and the DNA libraries were constructed using Nextera-XT DNA preparation kit and paired-end sequencing was performed on the Illumina HiSeq2500 (Illumina Inc., San Diego, CA) using a 500-cycle MiSeq reagent kit. A total of 4,468,030 reads were generated. Reads were *de novo* assembled using Velvet (9) which assembled them into 176 contigs ≥200 bp. The combined length of contigs is 4.90 Mbp with a G+C content of 51.82% and *N*_50_ value of 81.8 kbp. The contigs were ordered with Mauve (10) using the *Salmonella* LT2 genome as reference and coding sequences were predicted with prodigal (11). A total of 4,562 coding sequences (≥50 amino acids) were predicted within the genome. Signal peptide, clustered regularly interspaced short palindromic repeat (CRISPR) elements and resistance genes were predicted using signalp (12), CRISPR (13), and ARG-ANNOT (14), respectively. We identified signal peptides in 461 genes, and two CRISPR loci in the contigs. We identified one cryptic aac6-Iy and three active antibiotic resistance genes encoding resistance to gentamicin (*aac*[3]-*Iva*), tetracycline (*tetB*), and sulfisoxazole (sulI) antibiotics, which was validated through susceptibility testing. We have also identified a hygromycin resistance gene (*aph*[4]-*Ia*), which was not confirmed phenotypically. The information generated from the analysis of the genomes have helped predict phenotypic resistance and future comparative analyses will improve our understanding of genome evolution and multidrug resistance in *Salmonella*.

### Accession number(s).

The genome sequence of *Salmonella enterica* subsp. *enterica* serovar Putten strain CRJJGF_00159 has been deposited in the GenBank database (NCBI) under the accession number JQYK00000000. This paper describes the first version of the genome, JQYK00000000.1.
